# Re-Examining of Moffitt’s Theory of Delinquency through Agent Based Modeling

**DOI:** 10.1371/journal.pone.0126752

**Published:** 2015-06-10

**Authors:** Jia Ning Leaw, Rebecca P. Ang, Vivien S. Huan, Wei Teng Chan, Siew Ann Cheong

**Affiliations:** 1 Psychological Studies Academic Group, National Institute of Education, Nanyang Technological University, 1 Nanyang Walk, Singapore 637616; 2 Division of Physics and Applied Physics, School of Physical and Mathematical Sciences, Nanyang Technological University, 21 Nanyang Link, Singapore 637371; 3 Complexity Institute, Nanyang Technological University, 60 Nanyang View, Singapore 639673; Wake Forest School of Medicine, UNITED STATES

## Abstract

Moffitt’s theory of delinquency suggests that at-risk youths can be divided into two groups, the adolescence- limited group and the life-course-persistent group, predetermined at a young age, and social interactions between these two groups become important during the adolescent years. We built an agent-based model based on the microscopic interactions Moffitt described: (i) a maturity gap that dictates (ii) the cost and reward of antisocial behavior, and (iii) agents imitating the antisocial behaviors of others more successful than themselves, to find indeed the two groups emerging in our simulations. Moreover, through an intervention simulation where we moved selected agents from one social network to another, we also found that the social network plays an important role in shaping the life course outcome.

## Introduction

Juvenile delinquency is prevalent in cities [1, 2], perhaps due to the fact that cities create high social inequality, where the rich gets richer, while the poor remains poor [3, 4]. Neither social inequality nor delinquency is desirable, and both carry social costs beyond economic terms. As we struggle to find effective solutions to the problem of social inequality, which may in turn solve the problem of delinquency, the world rapidly urbanizes. It is projected that by 2050, 67.2% of the world’s population will live in cities[5]. Therefore, if we do not start finding solutions soon, the problem of juvenile delinquency will become ever more critical as we approach 2050. Another reason for urgency is the increasing likelihood that the socially disenfranchised delinquents may feed into religious self-radicalisation [6–8].

To start addressing the problem of delinquency, we invariably look first at Terrie Moffitt’s widely used taxonomy on youths’ antisocial behavior [9]. She categorizes at-risk youths into life-course-persistent and adolescence-limited groups. In the psychology literature, antisocial behaviors refer broadly to behaviors that go against accepted social norms [10–13]. Within this context, the life-course-persistent group exhibit antisocial behaviors since early childhood, and these persist throughout their entire life course. Moffitt suggested that such life-course-persistent antisocial behavior is mainly due to the neuropsychological impairment suffered at an early age. In contrast, the adolescence-limited group starts life in a typical fashion, and only begins to behave antisocially when they encounter a maturity gap, which is the perceived gap between the biological age and social age. This gap arises when their needs for freedom, material goods and sexual contacts are growing during their puberty period, but the society still considers them as underage and imposes various restrictions. At this stage in their life, the adolescence-limited individuals often perceive their antisocial life-course-persistent peers as coping better with the maturity gap. This motivates the former to mimic the antisocial behaviors of the latter.

**Fig 1:**
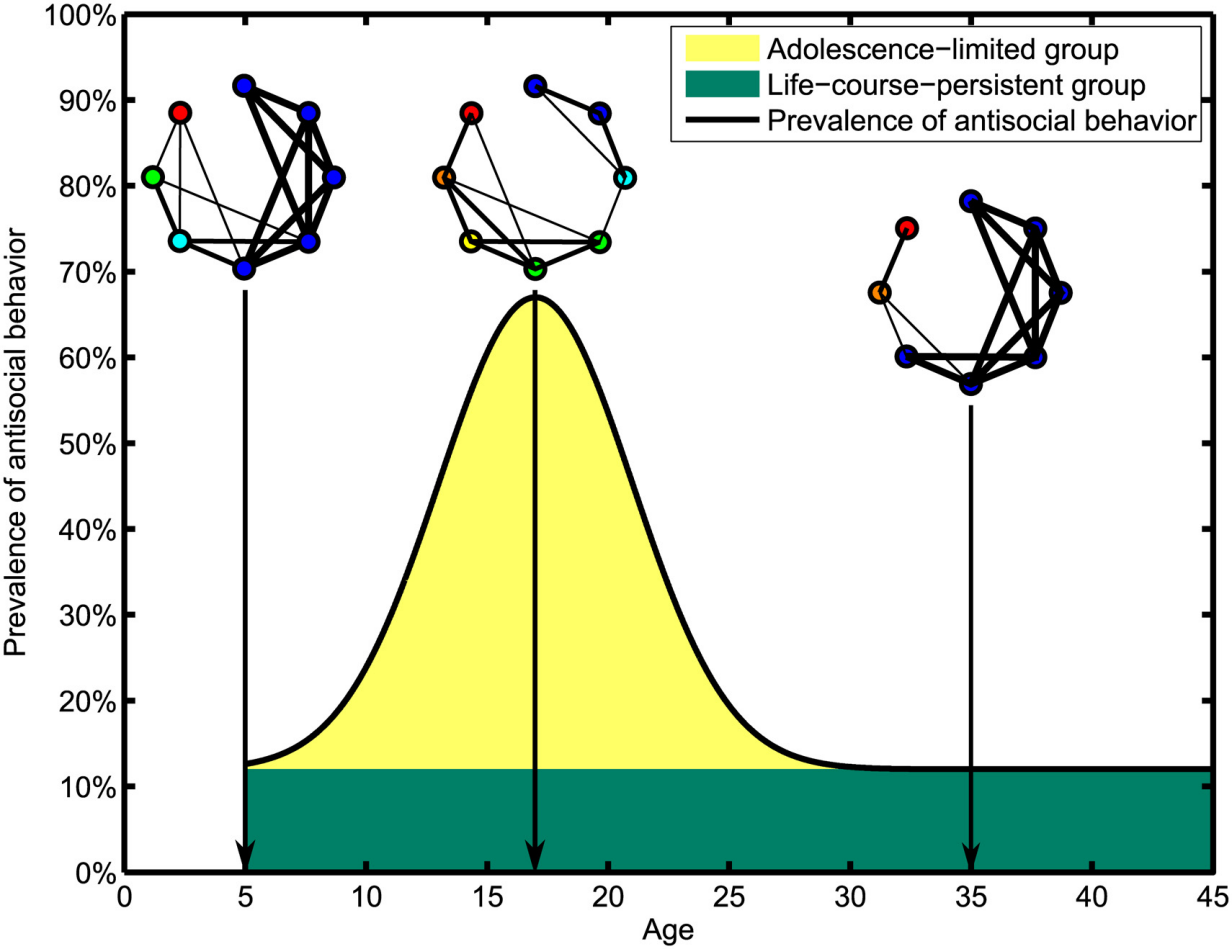
Moffitt’s illustration of the prevalence of antisocial behavior as a function of age, redrawn from Fig 3 in Ref. [9]. The black curve gives the *qualitative* prevalence of antisocial behavior among youths. In Moffitt’s theory, youths below the black curve are classified into two groups, life-course-persistent youths who remain antisocial throughout their life course, and adolescence-limited youths who behave antisocially only during their adolescent period. Also shown are typical peer network structures of the same eight individuals at different life stages, where blue nodes represent pro-social individuals and red nodes represent antisocial individuals. Green nodes represent marginally pro-social individuals while yellow nodes represent marginally antisocial. Thick links between nodes indicate frequent/strong contacts between these individuals, whereas thin links between other nodes indicate infrequent/weak contacts between these other individuals. In this figure, we illustrate how their dispositions and social structure change as a result of individuals mimicking others more antisocial than themselves during the maturity gap, and individuals more pro-social than themselves after the maturity gap. This social mimicry causes the social network to change from one organized around the pro-social individuals at around age 5 to one organized around the antisocial individuals at around age 17, and thereafter back to one organized around the pro-social individuals in adulthood.

When Moffitt developed this theory, she pointed out a few phenomena which we would like to highlight here. First, evidence shows that when youths grow up from early childhood to adolescence, the prevalence of antisocial behavior and criminal offences among youths increase with age (see Fig 1). During this transition, the life-course-persistent youths shift from peripheral to more influential positions in the peer social network. Their psychopathological behavior in childhood becomes normative, and the object of imitation by their adolescence-limited peers. Adolescence-limited youths, on the other hand, feel the need to exhibit antisocial behaviors to lessen the psychological burden they experience from the maturity gap. However, when adolescence ends, the trend reverses and both antisocial behaviors and criminal offences become less prevalent as the youths reach adulthood. After assuming legitimate adult roles and attaining adult privileges, the maturity gap is closed, and the adolescence-limited youths have no further need to behave antisocially. Moreover, behaving antisocially will also diminish their past achievements or jeopardize their future goals. With rewards turning into costs, the adolescence-limited group will quit behaving antisocially. In comparison, due to their long histories of antisocial behaviors, the life-course-persistent youths find few options for change, and thus they are more likely to remain antisocial.

In this paper, we build a complex agent network model incorporating the microscopic interactions: (i) a maturity gap that dictates (ii) the cost and reward of antisocial behavior, and (iii) agents imitating the antisocial behaviors of others more successful than themselves described in Moffitt’s theory. Complex networks have been the subject of intense studies over the past decade [14–16], and have been used as the basis for understanding the brain [17, 18], molecular biology [19–21], technology [22, 23], and society [24–27]. More recently, agent-based models and complex network models have been combined to give complex agent network models [28–30]. In agent-based models, we model the behaviors of discrete and autonomous constituents of a complex system, and their interactions with each other, with the expressed goal of studying emergent phenomena [31, 32]. In the agent-based modeling literature, an ‘agent’ is usually defined as an autonomous component of a heterogeneous system, capable of pro-active and reactive decision making based on its perceptions and interactions with other agents, and adapting to its environment [33–35]. Computer scientists go one step further to define an ‘agent’ to be “an encapsulated computer system that is situated in some environment and that is capable of flexible, autonomous action in that environment in order to meet its design objectives” [36]. However, one also finds in the agent-based modeling literature simpler entities that are accepted as ‘agents’, for example, in Schelling’s segregation model [37], as well as Epstein and Axtell’s sugarscape model [38]. We also find more sophisticated ‘agents’ that are not encapsulated, for example, Helbing’s social force model [39, 40]. Within this broad spectrum of models that are accepted as agent-based, the ‘agents’ in our model are recognizably autonomous and heterogeneous. They interact with other agents, and adapt to each other, but are conceptually closest to agents in the social force model, i.e. they are not encapsulated software objects, and have continuous internal states whose time evolution are governed by equations (as opposed to discrete internal states whose changes are governed by rules). There is however, one fundamental conceptual difference between the social force model (and similar equation-based agent-based models) and our complex agent network model: in addition to state variables associated only with the agents, we also have state variables associated with the interactions between agents.

Our paper is organized as follows. In the Materials and Methods section, we describe our model in detail. In particular, we explain how we start our simulations with pre-adolescent agents with a range of antisocial levels. We also explain how we initialize the simulations so that at this stage in their life course, a pro-social agent has stronger links than an antisocial agent. A pro-social agent thus occupies a more central position in the peer network while an antisocial agent finds itself in the periphery of the peer network. We then explain how we make agents imitate other agents that have higher antisocial levels than themselves during the maturity gap stage of the simulation, and how after this maturity gap is closed when the agents reach adulthood, agents go back to imitating more social agents. Finally, we describe how we perform an intervention analysis where we transplant an agent that is borderline life-course-persistent on its native peer network to a more pro-social peer network to test how effective the intervention would be. In the Results Section, we show how the life-course-persistent agents shift from periphery to the center of the network during the maturity gap. We also show that two groups of agents, life-course-persistent and adolescence-limited, naturally emerge from our model after the maturity gap closes. No initial classification into two groups was necessary. Since life-course-persistent delinquency is not necessarily predetermined in early childhood, we naturally ask whether we could intervene and change the natural progression of things for one or more life-course-persistent individuals? Indeed, we find in the intervention simulations that most of the transplanted agents were found to have lower final antisocial levels. In the Discussion Section we discuss the implications of our simulations on how we view life-course-persistent delinquency, and the potential efficacy of interventions.

## Materials and Methods

### Simulation Model

Our model contains a total of *N* agents representing the youths. We associate an antisocial level *e*_*i*_(*t*), that changes at every time step *t*, to each of the agents *i*. The initial antisocial levels of the agents are random numbers normally distributed with mean 0.5 and standard deviation 0.1. To ensure that these antisocial levels are within [0, 1], we redraw if a random number falls outside this range. Then we construct a fully-connected but weighted network. We assign a connection strength *A*_*ij*,unadjusted_ for the bidirectional link between each pair of agents *i* and *j*. The connection strengths are also initialized as uniformly distributed random number between 0 and 1. To agree with the observation that the life-course-persistent group is at the periphery of the friendship network initially, we need to adjust the connection strengths. This means that the lower the antisocial level of an agent, the stronger the connection he has with the other agents. To make this so, we multiply the connection strength between agents *i* and *j* by the prosocial levels, *r*_*i*_ = 1 − *e*_*i*_(*t* = 0) and *r*_*j*_ = 1 − *e*_*j*_(*t* = 0). This gives the adjusted connection strengths *A*_*ij*_ = *r*_*i*_ *r*_*j*_ *A*_*ij*,unadjusted_. Since the antisocial level *e*_*i*_ and the prosocial level *r*_*i*_ are values between zero and one, the multiplication of *r*_*i*_ and *r*_*j*_ serves to rescale the connection strength. This rescaling is done once at *t* = 0, and never done again in our simulations, so that *r*_*i*_ = 1 − *e*_*i*_(*t* = 0) represents the intrinsic prosocial level of agent *i*.

At each time step, agents affect each other’s antisocial levels according to their connection strengths. The change in the antisocial level of agent *i* due to agent *j* is proportional to the connection strength *A*_*ij*_ and the difference between the antisocial levels of agent *i* and agent *j*. We introduce the proportionality constant *c* to prevent the antisocial levels from changing too abruptly. Mathematically this reads

$$\begin{array}{c} d_{ij}\left(t\right)=cA_{ij}\left(t\right)(e_{j}\left(t\right)-e_{i}\left(t\right)),\;j\neq i, \end{array}$$ (1)

where *d*_*ij*_ is the change in the antisocial level of agent *i* contributed by agent *j*. With this interactions alone, however, the simulation will only end up at a state where all the agents have the same antisocial levels.

In Moffitt’s theory, youths become antisocial depending on the reward and cost associated with such behaviors. As mentioned in the Introduction, the reward of antisocial behavior is greater than the cost when youths are faced with the maturity gap. Once they reach adulthood, the cost of antisocial behaviors become even higher. We therefore introduce two variables, the reward *a* and the cost *b*, to each of the agents. These variables keep track of the tendency for agents to behave antisocially, and also decide whom the agents would befriend. Similar to the antisocial level and the connection strength, we restrict the values of *a* and *b* between 0 and 1.

To model an intensifying maturity gap experienced by youth as they grow up, we let the reward *a* increase with age until they reach a turning point *t*_*p*_. After *t*_*p*_, the reward for antisocial behaviors should slow down or stop increasing. For simplicity and also because of the lack of information from the real world, we model *a* to increase linearly before *t*_*p*_, and remains constant after *t*_*p*_. We allow the rates at which *a* increase during adolescence to be different for different agents, such that the rates are inversely proportional to their intrinsic prosocial level *r*_*i*_ = 1 − *e*_*i*_(*t* = 0). We also introduce a proportionality factor of *γ*, so that *a* would not increase too fast for 1/*r*_*i*_ > 1. If *a* still reach 1 before *t*_*p*_, it will remain at *a* = 1 thereafter. Mathematically, this reads

$$\begin{array}{c} a_{i}\left(t\right)=\min\;(\{\frac{\gamma }{r_{i}}\frac{t}{T},\frac{\gamma }{r_{i}}\frac{t_{p}}{T},1\}). \end{array}$$ (2)

Besides rewards, youths will also experience a “commitment cost” from antisocial behaviors. According to Moffitt’s theory, this cost arising from behaving antisocially is especially high when they start working. However, for youths that cannot find a proper job anyway because of their criminal records, then the cost is lower. Therefore, the cost *b* is assumed to depend on (1) the agent’s age, and (2) the agent’s antisocial history. In line with Moffitt’s theory, we would like the cost to increase sharply when the agents are ready to assume adult roles at *t*_*p*_. We therefore chose *b* to be proportional to the sigmoidal function,

$$\begin{array}{c} f\left(t\right)=\frac{1}{e^{-(t-t_{p})/k}+1}, \end{array}$$ (3)

where *k* is a simulation parameter that control the steepness of the sigmoidal curve. Since *b* is also related to the antisocial history, we define the antisocial history as the average antisocial level of an agent,

$$\begin{array}{c} h_{i}\left(t\right)=\frac{1}{t}\sum_{t^{\prime }=0}^{t}e_{i}\left(t^{\prime }\right). \end{array}$$ (4)

up to the time step where *b* is concerned. Since the cost decreases as *h*_*i*_ increases, we can say that the cost is proportional to the prosocial history (1−*h*_*i*_(*t*)) instead. The cost is therefore the product of the sigmoidal function and the prosocial history,

$$\begin{array}{c} b_{i}\left(t\right)=(1-\frac{\sum_{t^{\prime }=0}^{t}e_{i}\left(t^{\prime }\right)}{t})\;\frac{1}{e^{-(t-t_{p})/k}+1}. \end{array}$$ (5)

From the definitions of the reward *a*_*i*_(*t*) and the cost *b*_*i*_(*t*) given above, the net reward *g*_*i*_(*t*) = *a*_*i*_(*t*)−*b*_*i*_(*t*) falls within the range (−1,1). Together with *d*_*i*_(*t*) = ∑_*j*_ *d*_*ij*_(*t*), these variables will decide if the antisocial level of agent *i* increase or decrease at time step *t*. *d*_*i*_ corresponds to the contribution from peer influence, while *g*_*i*_ is the agent’s own tendency to increase or decrease *e*_*i*_. If *g*_*i*_ > 0, the agent receive a net reward from behaving antisocially, therefore, his antisocial level will increase. In addition, the larger the net reward, the agent will behave more antisocially. Thus, we set the increment of the antisocial level to be proportional to *g*_*i*_. However, when *g*_*i*_ > 0 we do not allow *e*_*i*_ to grow smaller than zero or larger than one. This means that the increment should be just a fraction of 1−*e*_*i*_(*t*) at time *t*. On the other hand, when the cost is larger than the reward, *g*_*i*_ < 0 and agent *i* will lower his antisocial level. In this case, we have to restrict *e*_*i*_ from going below zero. To do that, we lower *e*_*i*_ by ∣*g*_*i*_∣*e*_*i*_, following the argument that the larger ∣*g*_*i*_∣ is, the larger the decrement. Combining the cases of *g*_*i*_ > 0 and *g*_*i*_ < 0, we have the self contribution to the change in antisocial level

$$\begin{array}{c} d_{ii}\left(t\right)=\{\begin{array}{cc} g_{i}\left(t\right)(1-e_{i}\left(t\right)), & \text{if}\;g_{i}\left(t\right)\geq 0;\\ g_{i}\left(t\right)e_{i}\left(t\right), & \text{if}\;g_{i}\left(t\right)<0. \end{array} \end{array}$$ (6)

The total change of the antisocial level of agent *i* at time step *t* is, therefore, the average contribution from the other agents and agent *i* himself, and

$$\begin{array}{c} e_{i}\left(t+1\right)=e_{i}\left(t\right)+\frac{\sum_{j}d_{ij}\left(t\right)}{N}. \end{array}$$ (7)

Besides updating the antisocial levels, we also update the connections *A*_*ij*_ among the agents. We assume that agents with positive net rewards tend to connect to agents with higher antisocial level to mimic their behavior, while agents with negative net rewards will do the same to their peers with lower antisocial levels. However, we also assume that agent *i* will not mimic agent *j* if their antisocial levels are too different. This is because in the real world, people do not normally copy the actions of those who are too different from themselves. In our model, we prohibit agents from copying each other if the difference between their antisocial levels is larger than a gap Δ*e*. Else, if the difference between antisocial levels of agent *i* and agent *j* is less than Δ*e*, and if the net reward of agent *i* is positive (negative) with agent *j* more (less) antisocial than agent *i*, then the connection *A*_*ij*_ is strengthened by an amount proportional to: (1) the absolute value of net reward of agent *i*, ∣*g*_*i*_∣, (2) the difference between antisocial levels of agent *i* and agent *j*, ∣*e*_*j*_−*e*_*i*_∣, and (3) the gap between the current connection strength *A*_*ij*_(*t*) and 1.

The reason for (1) should be obvious: the higher the net reward *g*_*i*_ > 0, the more eager agent *i* is to mimic the antisocial behavior of agent *j*, so as to increase this net reward further. Conversely, the more negative the net reward *g*_*i*_ < 0, the greater the cost of antisocial behavior is to agent *i*, and the more willing he is to copy the less antisocial agent *j*. Also, agent *i* has little to gain from imitating an agent whose antisocial level is more or less the same as his, because the rewards and costs are very similar for the two agents. On the other hand, there is more to gain by imitating an agent with a very different antisocial level. Hence the condition (2). Finally, in (3) we require also that the change in the connection strength has to be proportional to the gap between the current connection strength and the maximum connection strength of 1. This is to ensure that the connection strength will not exceed 1 as it grows. In addition to these three factors, we introduce a proportionality constant of 1/Δ*e* to compensate for our restriction that the imitation occur only within a window of Δ*e*, so that the connection would not grow too slowly.

We also allow the connections to weaken. This is done in a way that mirrors how connections are strengthened, that is, the weakening of the connection is proportional to the net rewards of the influenced agent, and the difference in antisocial levels between the influencing and the influenced agents. The reduction is also proportional to gap between current connection strength and zero in this case. We also set the proportionality factor to be 1/Δ*e*. To summarize, the change of the connection strength is governed by the equations

$$\begin{array}{c} A_{ij}\left(t+1\right)=\{\begin{array}{cc} A_{ij}\left(t\right)+\Delta A_{ij}\left(t\right)(1-A_{ij}\left(t\right)), & \text{if}\;\Delta A_{ij}\left(t\right)\geq 0;\\ A_{ij}\left(t\right)+\Delta A_{ij}\left(t\right)(A_{ij}\left(t\right)-0), & \text{if}\;\Delta A_{ij}\left(t\right)<0, \end{array} \end{array}$$ (8)

where

$$\begin{array}{c} \Delta A_{ij}\left(t\right)=\{\begin{array}{cc} 0, & \text{if}\;|e_{j}\left(t\right)-e_{i}\left(t\right)|\geq \Delta e;\\ \frac{g_{i}\left(t\right)}{\Delta e}(e_{j}\left(t\right)-e_{i}\left(t\right)), & \text{if}\;|e_{j}\left(t\right)-e_{i}\left(t\right)|<\Delta e. \end{array} \end{array}$$ (9)

To summarize, to simulate the model, we:

1. calculate for each agent *i* its history of antisocial level up till time *t*, $h_{i}(t)=\sum_{t^{\prime }=0}^{t}e_{i}(t^{\prime })$, according to Eq (4);
2. calculate for each agent *i* the influences of its peers *j* ≠ *i*, *d*_*ij*_(*t*) = *cA*_*ij*_(*t*)(*e*_*j*_(*t*)−*e*_*i*_(*t*)), according to Eq (1);
3. calculate for each agent *i* the reward for antisocial behavior, $a_{i}(t)=\text{min}\left(\left\{\frac{\gamma }{r_{i}}\frac{t}{T},\frac{\gamma }{r_{i}}\frac{t_{p}}{T},1\right\}\right)$, according to Eq (2), where *r*_*i*_ = 1−*e*_*i*_(*t* = 0) is its intrinsic prosocial level;
4. calculate for each agent *i* the cost for antisocial behavior, $b_{i}(t)=\left(1-h_{i}(t)\right)\frac{1}{e^{-(t-t_{p})/k}+1}$, according to Eq (5);
5. calculate for each agent *i* the net reward *g*_*i*_(*t*) = *a*_*i*_(*t*)−*b*_*i*_(*t*);
6. update the network weights *A*_*ij*_ according to Eqs (8) and (9);
7. update the antisocial level of each agent *i*, $e_{i}(t+1)=e_{i}(t)+\frac{1}{N}\sum_{j=1}^{N}d_{ij}(t)$, according to Eq (7), where *d*_*ii*_(*t*) in Eq (6) is the self contribution to the change in antisocial level.

### Parameters

In our simulations, we set *N* = 30, which is roughly the number of youths in a small class. We chose this instead of a larger number of agents because a school-going child/adolescent spends a significant amount of time in school, and thus classroom interactions are very important, perhaps even critical to his/her propensity to offend. Also, outside of the classroom, adolescents may interact with contacts that are not shared with their classmates, and not of their ages. Thus if we chose to go beyond the classroom setting, the number of agents will become very large and very heterogeneous. More importantly, in the complex network sense, simulating only classroom interactions is akin to simulating the fully-connected core of the social network. We also set *k* = 20, *c* = 0.1, *γ* = 0.2,Δ*e* = 0.2 and *t*_*p*_ = 120. These values are chosen such that features in Moffitt’s theory are reproduced in our simulations, whereby a fraction of agents become life-course-persistent, and others adolescence-limited. We run each simulation for *T* = 300 time steps. Taking each time step to be a month, and assuming that the agents are seven years old when we start the simulations, the simulations span from seven years to thirty two years of age, with the turning point *t*_*p*_ at seventeen years old. The list of parameters, their roles, and their chosen values are summarized in Table 1.

**Table 1:** The list of parameters used in the simulations, their roles and their values. For the parameters *k*, *c*, Δ*e*, and *γ*, their values were increased and decreased by 50% (shown in parentheses) in the sensitivity analysis. The parameters *N*, *T*, and *t*_*p*_ were kept fixed for our simulations.

**Parameter**	**Role**	**Value**
*N*	Number of agents	30
*T*	Total number of time steps	300
*t*_*p*_	The turning time step when the agent start assuming adult roles	120
*k*	The steepness of the sigmoidal curve from which the commitment cost *b* is derived	20 (10, 30)
*c*	The proportionality constant that controls the rate of the agents imitating the antisocial behaviors from their peers	0.1 (0.05, 0.15)
Δ*e*	A window of antisocial level in which immitations occur	0.2 (0.1, 0.3)
*γ*	The rate of increase of the reward *a*	0.2 (0.1, 0.3)

### Sensitivity Analysis

In principle, the most useful agent-based models are calibrated against real-world data. However, the complex agent network model we describe in this paper is essentially a toy model intended for exploring which features of Moffitts dual taxonomy are necessary, and which features of the theory are emergent. We are not aiming for quantitative agreement and predictive power. More importantly, life course data that would allow us to calibrate our model is hard to come by, and generally insufficient. In particular, the cost and reward of antisocial behavior, as well as how strongly juveniles mimic each other can only be measured through very carefully designed surveys and interviews. Therefore, there is no easy way to get at the parameters *k*, *c*, and *γ*. Nevertheless, we performed sensitivity analysis for the parameters chosen in Table 1, to reassure ourselves that our model is plausible and well-behaved.

In this simulation study, the key macroscopic outcome that we monitor is the ratio of adolescence-limited agents to life-course-persistent agents at the end of the simulations. To test the sensitivity of this ratio to the parameter set that we have chosen, we performed the following test. Because the simulation outcome depends sensitively on the initial antisocial levels, we first obtain the distribution of the final proportion of adolescence-limited agents *F*(**p**_0_) from 1000 simulations for our parameter set, **p**_0_ = {*k* = 20, *c* = 0.1, *γ* = 0.2,Δ*e* = 0.2}, with random initial antisocial levels obtained using the initialization procedure described in the Materials and Methods Section. We then change each of the parameters by 50% to obtain

$$p_{k+}=p_{0}+\delta p_{k},\;p_{k-}=p_{0}-\delta p_{k},$$ (10)

$$p_{c+}=p_{0}+\delta p_{c},\;p_{c-}=p_{0}-\delta p_{c},$$ (11)

$$p_{\gamma +}=p_{0}+\delta p_{\gamma },\;p_{\gamma -}=p_{0}-\delta p_{\gamma },$$ (12)

$$p_{\Delta e+}=p_{0}+\delta p_{\Delta e},\;p_{\Delta e-}=p_{0}-\delta p_{\Delta e},$$ (13)

where

$$\delta p_{k}=\{10,0,0,0\},\;\delta p_{c}=\{0,0.05,0,0\},$$ (14)

$$\delta p_{\gamma }=\{0,0,0.1,0\},\;\delta p_{\Delta e}=\{0,0,0,0.1\}$$ (15)

are the 50% change from **p**_0_ for each of the parameters. For each **p**_*i*+_ and **p**_*i*−_, we run 1000 simulations, and obtain the distributions *F*(**p**_*i*+_) and *F*(**p**_*i*−_).

To quantify the sensitivity of the simulation outcomes to our choice of parameters, we make use of the Jensen-Shannon divergence (JSD) to measure the difference between two distributions. To introduce JSD, we need to first introduce another divergence called Kullback-Leibler divergence (KLD). For two probability distributions *F*_**p**_1__ and *F*_**p**_2__, KLD is defined as

$$\begin{array}{c} KLD(F_{p_{1}}\left|\right|F_{p_{2}})=\sum_{i}F_{p_{1}}\left(i\right)\ln(\frac{F_{p_{1}}\left(i\right)}{F_{p_{2}}\left(i\right)}), \end{array}$$ (16)

where *i* is the index for the bin of the discrete distributions, which is suppressed in our notation earlier to highlight the dependence of the distribution on the parameter set. For cases where *F*_**p**_2__(*i*) = 0, we set the argument of the logarithm function to 1. In other words, KLD is the expectation of the logarithmic difference between the two probability distributions, with the expectation taken using *F*_**p**_1__. KLD is normally used in the cases where we want to measure how *F*_**p**_1__ differs from the reference distribution *F*_**p**_2__. However, in our case, both *F*_**p**_1__ and *F*_**p**_2__ are not reference distributions. A third distribution *F*_*M*_ = (*F*_**p**_1__+*F*_**p**_2__)/2, which is the average between *F*_**p**_1__ and *F*_**p**_2__, is used as the reference instead. The JSD is then the average of the KLD of *F*_**p**_1__ from *F*_*M*_, and KLD of *F*_**p**_2__ from *F*_*M*_,

$$\begin{array}{c} JSD\;(F_{p_{1}},F_{p_{2}})=\frac{1}{2}\;(KLD\;(F_{p_{1}}\left|\right|F_{M})+KLD\;(F_{p_{1}}\left|\right|F_{M})). \end{array}$$ (17)

With the JSD as the difference between two distributions, the sensitivity of the outcome distributions to the parameter set is then the rate of change of the difference with respect to the parameter set. Thus, we use the gradient of the Jensen-Shannon divergence in the parameter space ∇_**p**_ *JSD*(*F*(**p**_0_), *F*(**p**)) as our measure of sensitivity. Since *JSD*(*F*(**p**_0_), *F*(**p**_0_)) = 0, and

$${\frac{\partial JSD\;(F\;(p_{0}),F\;(p))}{\partial p_{i}}|}_{p=p_{0}}=\lim_{\delta p_{i}=0}\frac{JSD\;(F\;(p_{0}),F\;(p_{0}+\delta p_{i}))-JSD\;(F\;(p_{0}),F\;(p_{0}))}{\delta p_{i}}$$ (18)

$$\begin{array}{c} \approx \frac{JSD\;(F\;(p_{0}),F\;(p_{i+}))}{\delta p_{i}}, \end{array}$$ (19)

we approximate the gradient as the forward difference

$$\begin{array}{c} \nabla_{p}JSD\;(F\;(p_{0}),F\;(p))\approx [\begin{array}{c} JSD\;(F\;(p_{0}),F\;(p_{k+}))/\delta p_{k}\\ JSD\;(F\;(p_{0}),F\;(p_{c+}))/\delta p_{c}\\ JSD\;(F\;(p_{0}),F\;(p_{\gamma +}))/\delta p_{\gamma }\\ JSD\;(F\;(p_{0}),F\;(p_{\Delta e+}))/\delta p_{\Delta e} \end{array}], \end{array}$$ (20)

or the backward difference

$$\begin{array}{c} {}[\begin{array}{c} JSD\;(F\;(p_{0}),F\;(p_{k-}))/\delta p_{k}\\ JSD\;(F\;(p_{0}),F\;(p_{c-}))/\delta p_{c}\\ JSD\;(F\;(p_{0}),F\;(p_{\gamma -}))/\delta p_{\gamma }\\ JSD\;(F\;(p_{0}),F\;(p_{\Delta e-}))/\delta p_{\Delta e} \end{array}]. \end{array}$$ (21)

### Intervention Analysis

**Fig 2:**
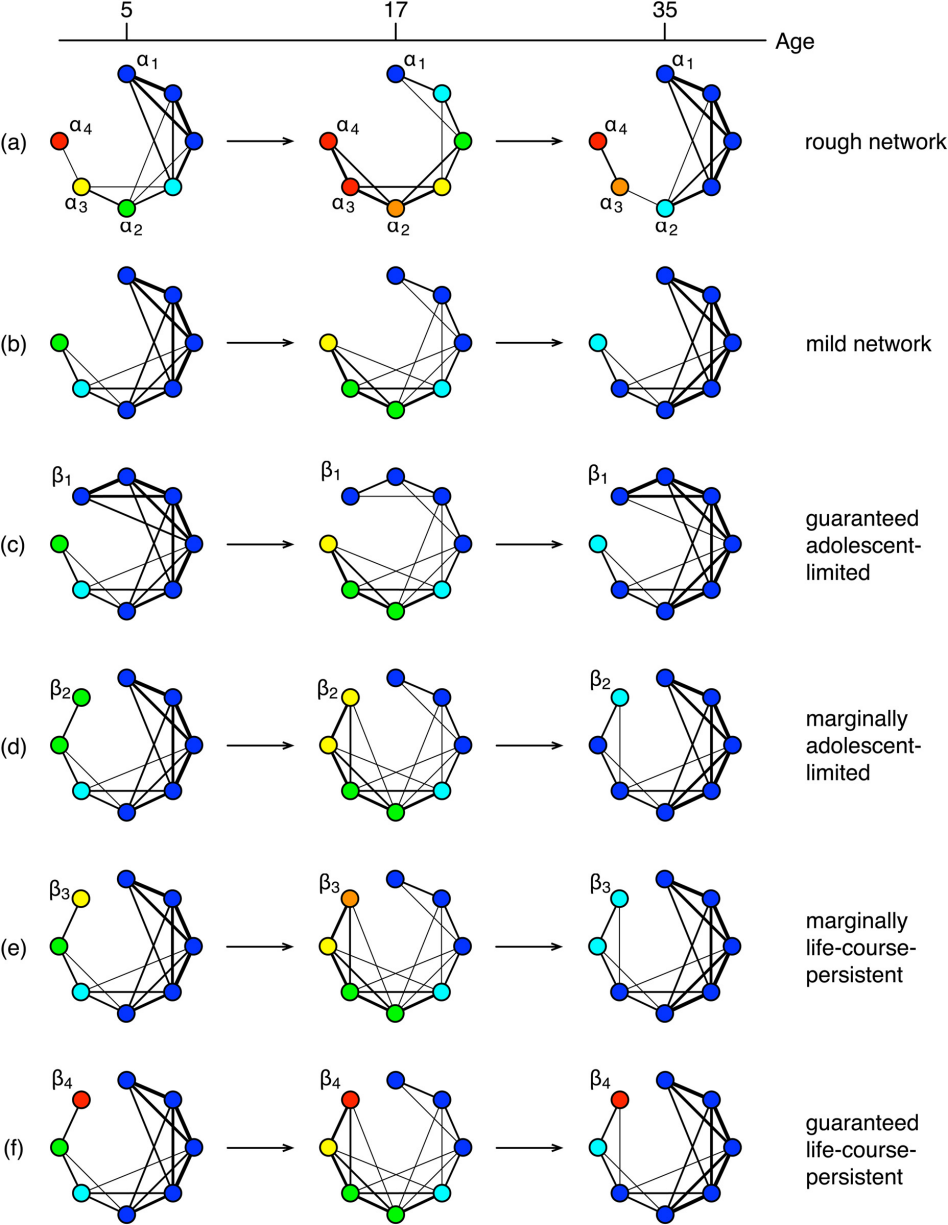
The peer network structure at age 5, age 17, and age 35 for a (a) *rough network*, (b) *mild network*, and various intervention scenarios ((c)-(f)). In this figure, blue agents have the lowest antisocial levels while red agents have highest antisocial levels. Cyan, green, yellow, and orange agents have increasingly higher antisocial levels. In our intervention analysis, we first run the simulations repeatedly using the parameters shown in Table 1, but with different initial antisocial levels for the *N* = 30 agents. Depending on the initial antisocial levels, the simulations ended up with different numbers of life-course-persistent agents. We then pair (a) 50 initial antisocial levels with at least five agents having antisocial levels higher than 0.5 at the end of the simulations (rough networks) up with (b) 50 initial antisocial levels with no agent having antisocial level higher than than 0.5 at the end of the simulations (mild networks) for each of the four scenarios we analyzed. In scenario (c), we identify the least antisocial agent *α*_1_ in the rough network, and add an agent *β*_1_ with the same initial antisocial level as *α*_1_ to the mild network to simulate. In scenario (d), we identify the agent *α*_2_ ending up as marginally adolescence-limited in the rough network, and add an agent *β*_2_ with the same initial antisocial level as *α*_2_ to the mild network to simulate. In scenario (e), we identify the agent *α*_3_ ending up as marginally life-course-persistent in the rough network, and add an agent *β*_3_ with the same initial antisocial level as *α*_3_ to the mild network to simulate. Finally, in scenario (f), we identify the most antisocial agent *α*_4_ in the rough network, and add an agent *β*_4_ with the same initial antisocial level as *α*_4_ to the mild network to simulate.

In this part of our study, we check if an agent who is borderline life-course-persistent will become adolescence-limited if he is moved to a milder social network. First, we run the simulations repeatedly until we find a set of initial antisocial levels where at the end of the simulation, there are at least five agents with antisocial levels higher than 0.5. This is our rough social network that produces a number of life-course-persistent agents (Fig 2A). We then run the simulations repeatedly again until we find a set of initial antisocial levels where at the end of the simulation, there is no agent with antisocial level higher than 0.5. This is our mild social network (Fig 2B). We repeat this process 50 times, to find 50 pairs of rough and mild social networks.

To properly measure the efficacy of our intervention measure, we select four agents from the rough network and add them to the mild network. The first of these agents (*α*_1_) is guaranteed to be adolescence-limited (even in the rough social network), because of his lowest antisocial level. The fourth of these agents (*α*_4_) is guaranteed to be life-course-persistent (even in the mild social network), because of his highest antisocial level. The second (*α*_2_) and third (*α*_3_) of these agents are marginally adolescence-limited and marginally life-course-persistent. The two marginal agents are selected using the fact that there is usually a big gap in the antisocial level between the life-course-persistent and adolescence-limited groups, and thus we can list all agents in ascending order of their antisocial levels, and select the two agents differ the most from each other, with the one with lower antisocial the borderline adolescence-limited agent, and the one with higher antisocial the borderline life-course-persistent agent. We transplant these agents into the milder network, one at a time (Fig 2C–2F).

According to Moffitt’s theory, a youth’s neuropsychological impairment is the intrinsic property that strongly influences whether he belongs to the life-course-persistent group or adolescence-limited group. In our simulation, the intrinsic property of an agent is his antisocial level. The reward and cost of an agent can be derived from his initial antisocial level, thus when we move agent *α* from one network to another, only the initial antisocial level needs to be transferred. To move the agent from the rough network to a mild network, we simply add an agent *β* to the mild network and set *β*’s initial antisocial level to *α*’s initial antisocial level. We randomly initialize *β*’s connections to the other agents using the algorithm described above. We then run this intervention simulation in the same way, but now with one more agent (*β*).

## Results

**Fig 3:**
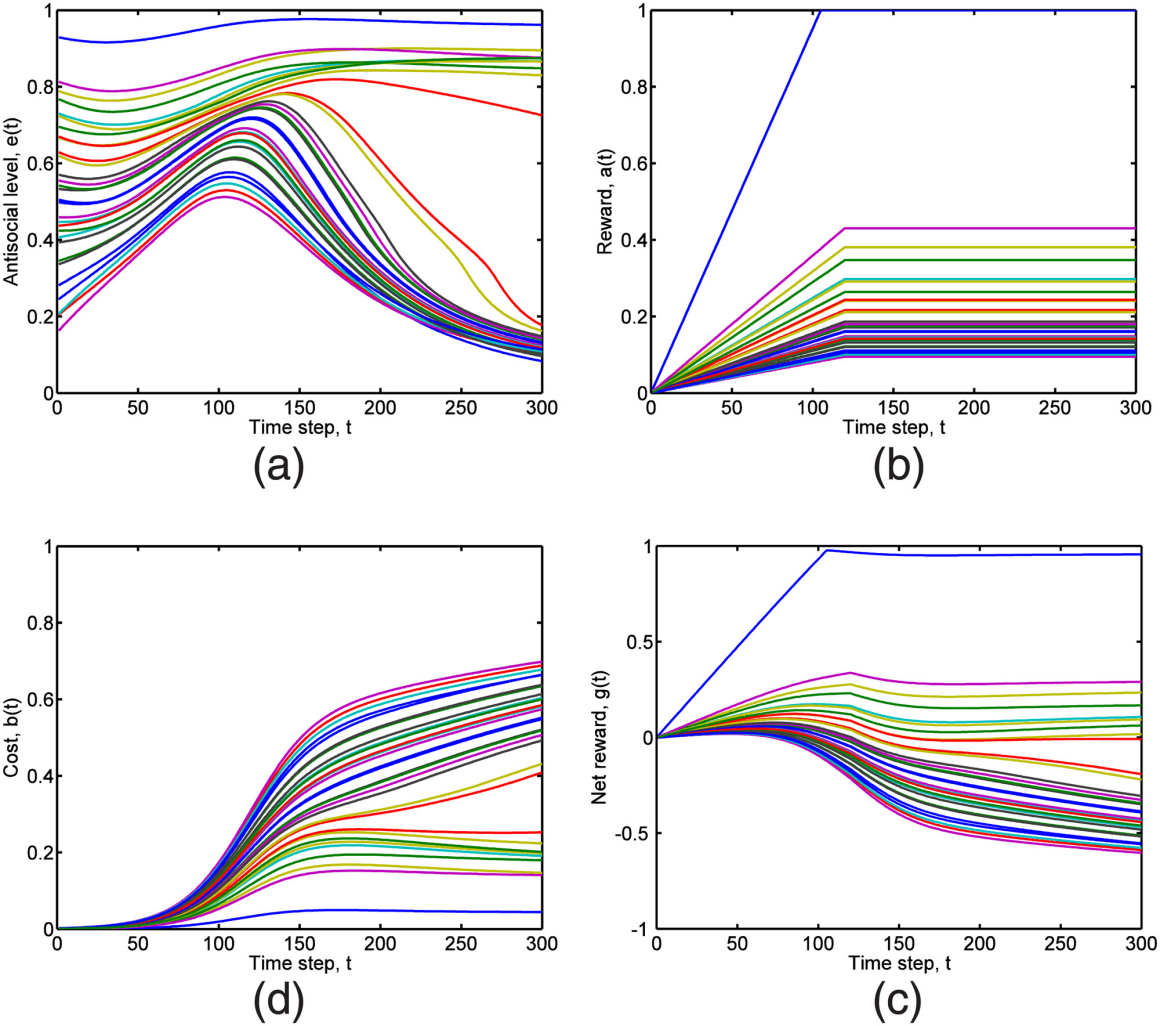
The (a) antisocial levels *e*_*i*_(*t*), (b) rewards *a*_*i*_(*t*), (c) costs *b*_*i*_(*t*), and (d) net rewards *g*_*i*_(*t*) of the 30 agents in a typical simulation. In (b), the rewards increase linearly with time in the maturity gap, to reach a different constant for different agents in adulthood. In (c), we see how the costs change with time for all 30 agents. The cost for a given agent is a not a simple sigmoid because his antisocial level changes with time. We also see a gap opening up between a group of agents with nearly constant cost and a group of agents with increasing cost near the end of the simulation. This gap is also seen in (d) the net reward, but is most pronounced in (a) the antisocial level.

**Fig 4:**
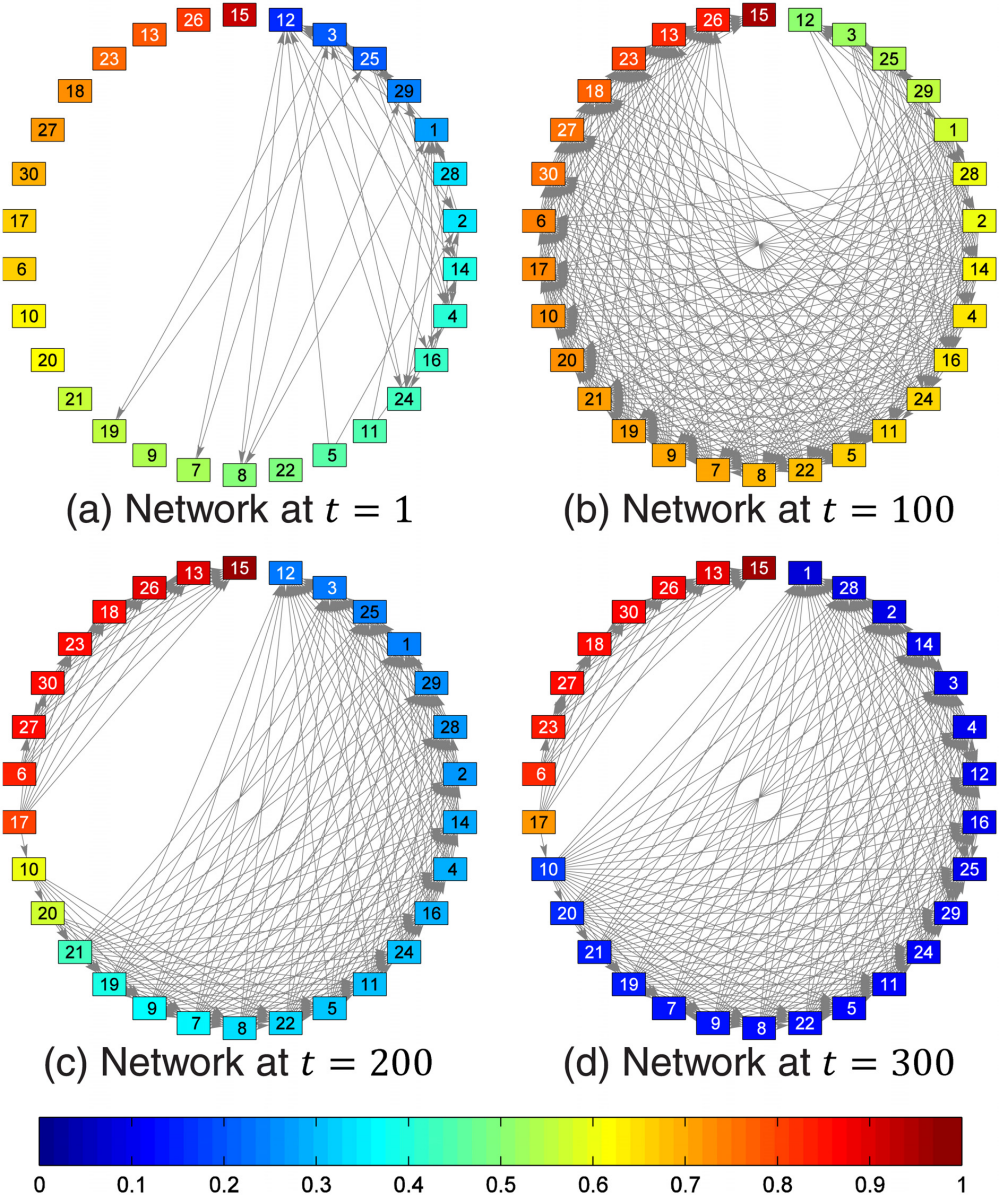
The peer network structure at (a) *t* = 1 (age 7), (b) *t* = 100 (age 15), (c) *t* = 200 (age 23), and (d) *t* = 300 (age 32). The boxes represent the agents, and the labels of the boxes are the indices of the agents. The agents are ordered clockwise from the least antisocial to the most antisocial, and the color of the boxes indicate the antisocial levels of the agents, according to the colorbar at the bottom. All agents are connected to each other, but here we show only the directed connections whose weights are larger than 0.3. In this figure, we see that (a) the initial connections at age 7 are strong only between pro-social agents, and agents mostly imitate agents less antisocial than themselves. During the maturity gap at (b) age 15, social mimicry amongst agents strengthens most connections, and agents mostly imitate agents more antisocial than themselves. After entering adulthood at (c) age 23, the agents split into two groups, a pro-social group where agents imitate those less antisocial than themselves, and an antisocial group where agents imitate those more antisocial than themselves. These two groups become more distinct as time goes on, as we can see in (d) at age 32.

We show the key variables in our model, the antisocial level, the reward, the cost, and the net reward as functions of time in Fig 3. In Fig 4, the peer networks at different times are shown. The networks are fully-connected and weighted, but for illustration purposes, only connections with weights larger than 0.3 are shown. Note that the value 0.3 is arbitrary and does not affect the dynamics of the simulations, but is chosen to best visualize the networks. The connections are also shown with equal thickness although they have different weights. This is because we would like to highlight the influential agents, and do not want the readers to be distracted by lines with different thicknesses. The influential agents are nodes with many arrows pointing to them.

At *t* = 100, agents with high antisocial levels become the most influential agents, with many arrows pointing toward them. This situation changes when the simulation reaches *t* = 200, where the agents have moved into the adult phases of the life course, and the maturity gap has closed. At this time step, two clusters are clearly seen, one associated with the life-course-persistent group, and the other, the adolescence-limited group. This outcome agrees with the dual taxonomy proposed by Moffitt.

**Fig 5:**
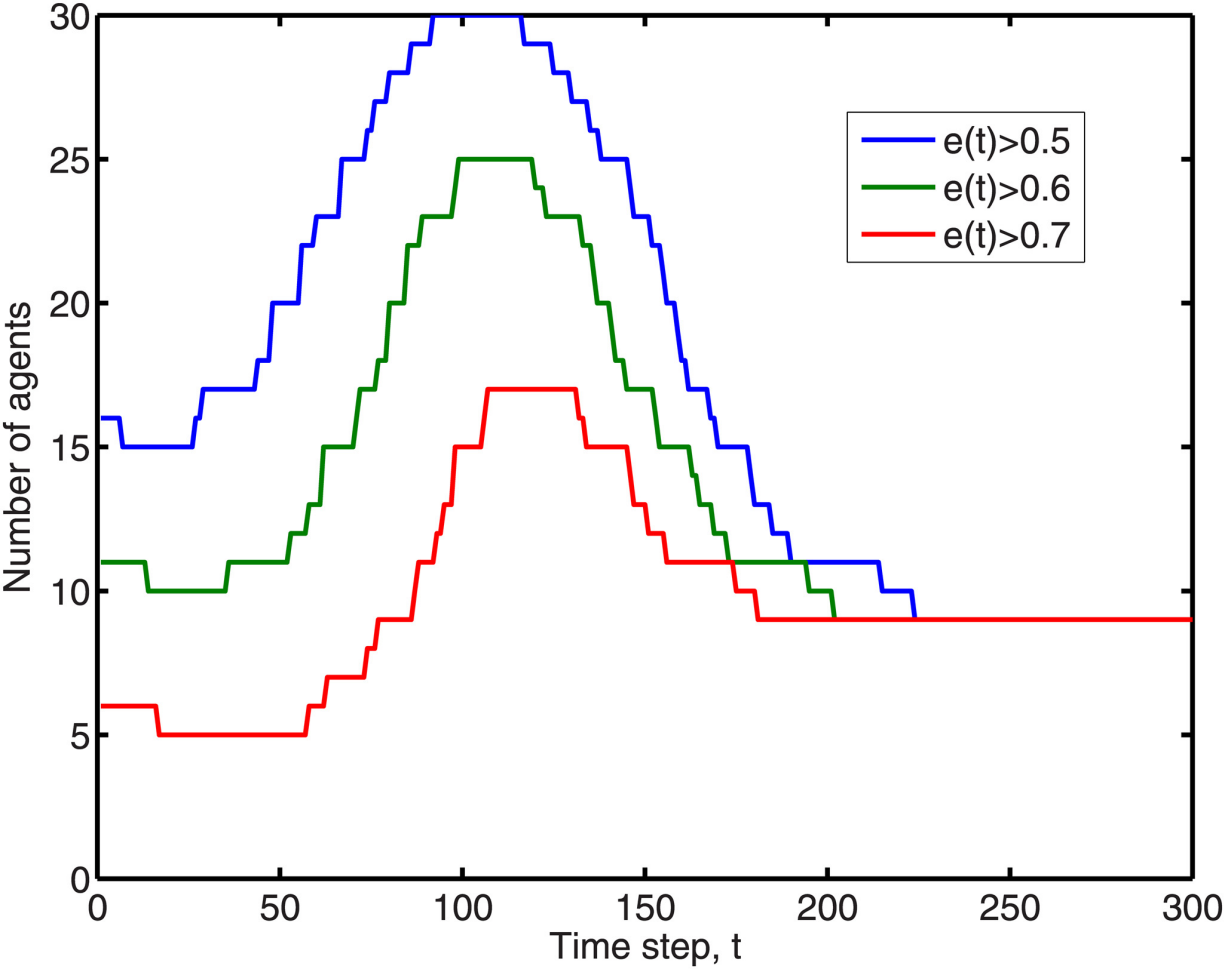
From Fig 4, we see that the pro-social group ended up with antisocial levels 0 < *e*_*i*_(*t*) < 0.5 while antisocial group ended up with antisocial levels 0.7 < *e*_*i*_(*t*) < 1. Therefore, we can use an antisocial level between 0.5 and 0.7 as the threshold for delinquency, and count the numbers of agents with antisocial levels larger than 0.5, 0.6 and 0.7 respectively, as they evolve over time. We see that whatever threshold we use, we end up with an offending rate qualitatively similar to the one sketched by Moffitt in Fig 1.

We also plot in Fig 5 the curves showing the number of agents with antisocial level larger than various thresholds over time. These curves resemble the features described in Moffitt’s theory, in that prevalence of the antisocial behavior is low at a very young age, peaks during the adolescence period, and decay again when the agents go into adulthood.

**Fig 6:**
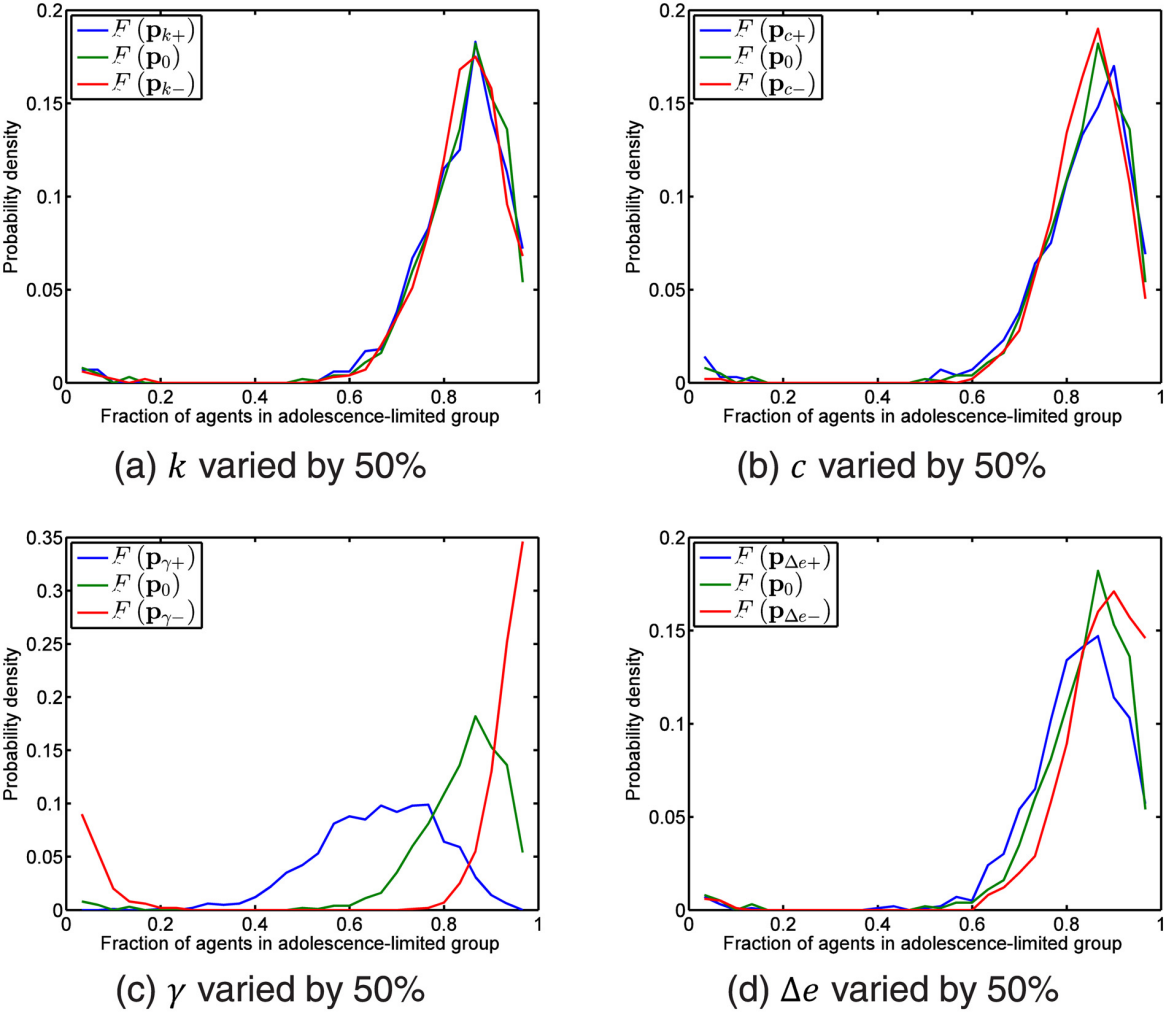
The distributions of the end-simulation adolescence-limited proportion when (a) *k* is increased or decreased by 50%, (b) *c* is increased or decreased by 50%, (c) *γ* is increased or decreased by 50%, and (d) Δ*e* is increased or decreased by 50%, compared against the benchmark distribution *F*(**p**_0_) for the parameters shown in Table 1. The bin size for binning the adolescence-limited fraction is 1/*N* where *N* = 30 is the number of agents in one simulation. As we can see, the simulation outcome is most sensitive to the parameter *γ*, which is the rate of increase of the reward for antisocial behavior. Our model is next most sensitive to Δ*e*, the range of antisocial levels over which social mimicry can occur. Our model is very insensitive to the parameters *k*, which is how steeply the cost change with antisocial level and *c*, which is the proportionality constant that limits how fast agents can imitate each other.

The results of our sensitivity analysis are shown in Fig 6. First, let us look at *F*(**p**_0_), the distribution of proportion of adolescence-limited agents at the end of the simulation using parameter values listed in Table 1. We tuned the model parameters to get about 80% of the agents to be adolescence-limited at the end of the simulation on average, so indeed, *F*(**p**_0_) is sharply peaked around 85%. However, the width of this peak is about 15%, and hence there is considerable variance in the simulation outcomes. Because of this variability, we chose to compare distributions instead of averages. We see that when *k*, *c*, and Δ*e* were changed by as much as 50%, there is little change to the distribution of final adolescence-limited fractions. When we change *γ* by 50%, however, the outcomes are significantly altered. When *γ* is reduced from 0.2 to 0.1, most simulations ended up with final adolescence-limited fraction larger than 0.8. In contrast, when *γ* is increased from 0.2 to 0.3, many simulations ended with a lower adolescence-limited fraction.

This sensitivity of the model to *γ* can be seen quantitatively from the forward difference approximation to the gradient of the JSD,

$$\begin{array}{c} {}[\begin{array}{c} JSD\;(F\;(p_{0}),F\;(p_{k+}))/\delta p_{k}\\ JSD\;(F\;(p_{0}),F\;(p_{c+}))/\delta p_{c}\\ JSD\;(F\;(p_{0}),F\;(p_{\gamma +}))/\delta p_{\gamma }\\ JSD\;(F\;(p_{0}),F\;(p_{\Delta e+}))/\delta p_{\Delta e} \end{array}]=[\begin{array}{c} 0.0004\\ 0.1297\\ 2.8089\\ 0.1004 \end{array}], \end{array}$$ (22)

or the backward difference approximation

$$\begin{array}{c} {}[\begin{array}{c} JSD\;(F\;(p_{0}),F\;(p_{k-}))/\delta p_{k}\\ JSD\;(F\;(p_{0}),F\;(p_{c-}))/\delta p_{c}\\ JSD\;(F\;(p_{0}),F\;(p_{\gamma -}))/\delta p_{\gamma }\\ JSD\;(F\;(p_{0}),F\;(p_{\Delta e-}))/\delta p_{\Delta e} \end{array}]=[\begin{array}{c} 0.0007\\ 0.1466\\ 2.4598\\ 0.2276 \end{array}]. \end{array}$$ (23)

Indeed, as we can tell by visual inspection of Fig 6, the simulation outcome is significantly more sensitive to *γ* than it is to the other three parameters. We therefore need only justify the choice of *γ* = 0.20. As we can see from Fig 6C, with this choice of *γ* we end up with predominantly 80% of the cohort being adolescence-limited. This is reasonable, as Moffitt believes that the proportion of life-course-persistent delinquents in the real world should be about 5%-10% [9].

**Fig 7:**
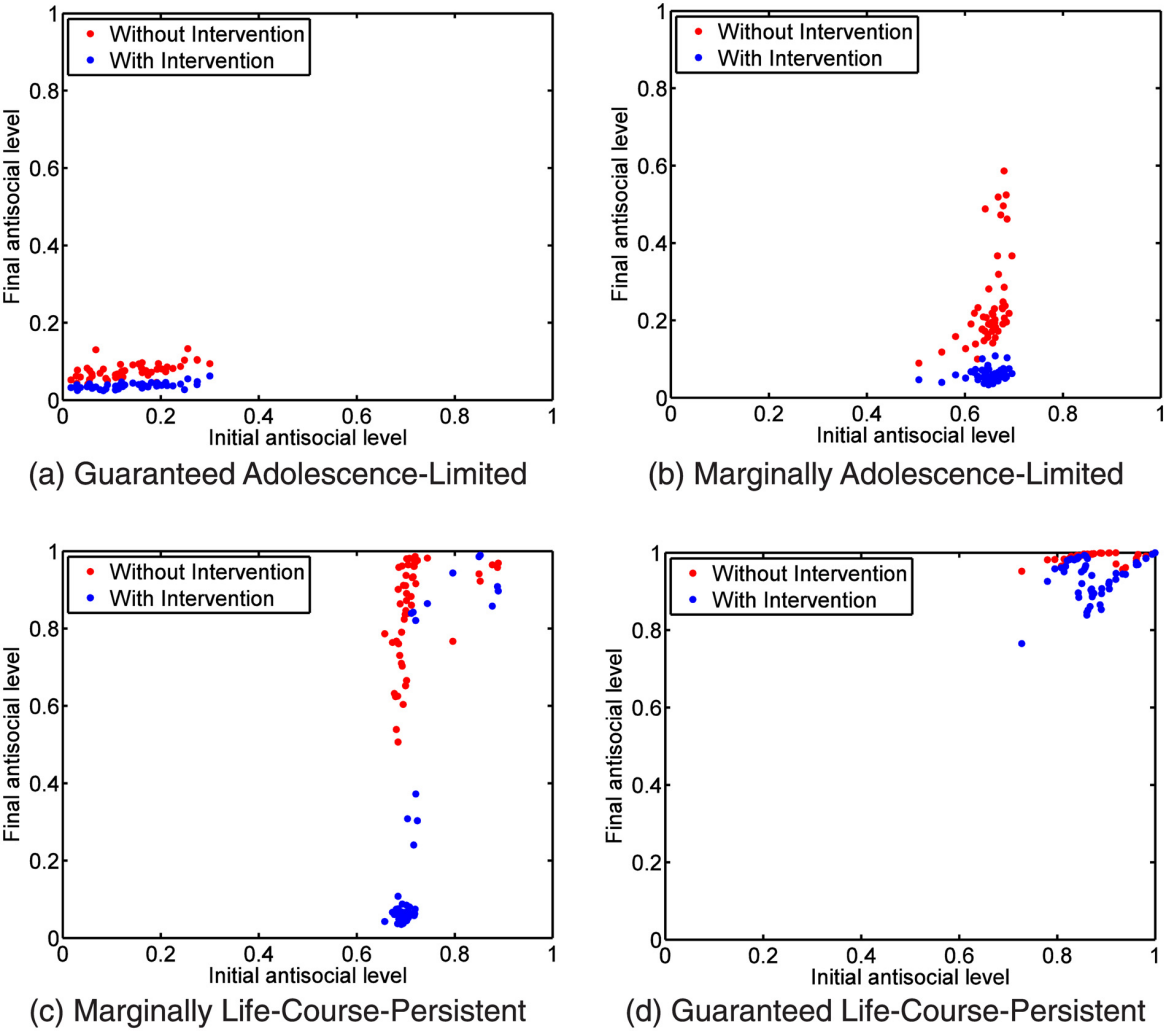
Intervention analysis comparing the final antisocial level of an agent in its native, rough network to his final antisocial level after he has been moved to a mild network. For each of the following four scenarios, we ran 50 simulations. In (a), a guaranteed adolescence-limited agent is moved from the rough network to the mild network, and we see that there is little change in his final antisocial level with or without intervention. In (b), a marginally adolescence-limited agent is moved from the rough network to the mild network, and we see a statistically significant reduction in his final antisocial level. In (c), a marginally life-course-persistent agent is moved from the rough network to the mild network. The reduction in final antisocial level is the largest in this scenario, even though there are cases where the intervention fails, and the agent remains life-course-persistent. Finally, in (d) we move a guaranteed life-course-persistent agent from the rough network to the mild network. Although in some cases we see reduction in the final antisocial level, the agent remains life-course-persistent.

Finally, in the intervention analysis, the final antisocial levels of the target agents in both scenarios are plotted against their initial antisocial level in Fig 7. We can see that generally the milder social networks reduce the antisocial levels of the transplanted agents. The intervention is most effective for the borderline life-course-persistent agent, with the antisocial levels reduced by 0.60 ± 0.32, followed by the borderline adolescence-limited agent, 0.18 ± 0.12. The guaranteed adolescence-limited and guaranteed life-course-persistent agents do not benefit much from the intervention, and their reductions in antisocial levels are only 0.038 ± 0.019 and 0.056 ± 0.053 respectively.

## Discussion

Despite the relative simplicity of our model, where we assumed only (i) the existence of a maturity gap that dictates the (ii) cost and reward for antisocial behaviors, and (iii) agents imitate the antisocial behavior of others more successful than themselves, we managed to reproduce key macroscopic outcomes described in Moffitt’s theory. First of all, we see that two groups of agents emerge after going through the maturity gap: one with high antisocial levels, and the other, low antisocial levels. These corresponds to Moffitt’s dual taxonomy of delinquent youths, where the group with high antisocial level is the life-course-persistent group while the group with low antisocial level is the adolescence-limited group. It is important to note the dual taxonomy was built into the model. As far as possible, we chose functional forms for the cost and reward based on how Moffitt describes them in qualitative terms. Like the cost and reward, social mimicry is also operative at all times during the simulation. We do not turn imitation on or off, or bias which other agents an agent imitates. The targets that an agent chose to imitate is decided entirely by the cost and reward, along with a restriction that agents do not imitate others too different from themselves. This last restriction is our choice, as Moffitt did not mention who can imitate whom, and by how much. The social mimicry consists of two parts: (i) an agent updates its antisocial level to become more similar to its target, and (ii) the strength of a connection between an agent and its target becomes stronger if the imitation results in positive net reward, and becomes weaker if the imitation results in negative net reward. Beyond these mechanistic rules in our model, we initialize the antisocial levels of the agents randomly, and the peer network structure to be biased against antisocial agents. We then ran the simulation. Moffitts dual taxonomy, as well as the periphery-to-center-to-periphery changes to where the antisocial agents are located on the peer networks is a bona fide emergent phenomenon in our simulations.

In 1993, Moffitt explained that in the modern day context, because of improved nutrition, healthcare, longer education and delayed entry into the workforce, adolescents find themselves biologically capable but socially and economically dependent on their families, and do not make many decisions of real import. Contemporary adolescents are thus trapped in what Moffitt terms as a ‘maturity gap’ between biological and social age. These adolescents perceive that their life-course-persistent counterparts, having entered delinquency much earlier, as having access to valued adult resources such as cars, drugs and sex, and so they would mimic the antisocial behavior and lifestyle in order to also access these resources and adult privileges [41, 42]. Secondly, we see that life-course-persistent agents, in Moffitt’s own words [9], “shift from peripheral to more influential positions in the peer social structure” during the adolescence period, which is when the time step *t* ≈ 100. Moffitt explained that this change is a natural consequence of the social mimicry by adolescence-limited individuals, and in Ref. [9], also cited empirical findings [43] that life-course-persistent individuals are not well integrated into the broader social network, but selectively seek out other antisocial individuals to connect to [44, 45]. We also see that our number of agents with antisocial behavior larger than some threshold versus time step plot agrees with Moffitt’s description that the prevalence of the antisocial behavior peaks during the adolescent period.

While our work is the first computational modeling test of Moffitt’s dual taxonomy, which she first proposed in 1993 [9] and thereafter reviewed in 2003 [46], there have been many empirical studies to validate her theory [47, 48]. There are other researchers who propose various configurations or groupings [49–51]—but the simplest building block for all these other variations would be Moffitt’s two groups. Different researchers also use different terminology—not all call them life-course-persistent versus adolescence-limited. They use different words like “high-level chronic offenders”, and so on that bear similarity to Moffitt’s two groups [52, 53]. Of course, there are also researchers who believe that the two groups are not as distinct as assumed in Moffitt’s theory [54, 55]. Moreover, they argue that every individual is different, and hence categorizing juvenile delinquents into two groups is too narrow and limiting and does not reflect reality. Within the context of this debate it is important to note that we ended up with Moffitt’s dual taxonomy without assuming that the agents started out as such. In fact, we started them out with a continuous spectrum of antisocial levels, have the agents mimic each other, and allow the cost and reward of antisocial behavior to reshape their interactions and social network. In the end, two quantitatively and qualitatively distinct groups of agents emerged in our simulations. This attests to the utility of agent-based modeling and simulations to generate insights into problems of human complexity such as juvenile delinquency.

Nevertheless, when translating Moffitt’s qualitative descriptions into equations and rules, even if we remain disciplined and model microscopic (causal) mechanisms but not macroscopic (possibly emergent) features, we need to make modeling choices. For example, the reward function is posited to increase with age until 18 years, thereafter becoming constant. This is a modeling choice. We could also have the reward function decrease after 18 years of age, or increase at a slower rate after 18 years. We were fortunate to have picked an interesting reward function, because it may be that some choices will not reproduce Moffitt’s dual taxonomy (unless it is explicitly built into the model). Different modeling choices where Moffitt’s dual taxonomy does emerge are also likely to lead to different final proportions of life-course-persistent delinquency. Therefore, unless we have much more data, perhaps from a highly intrusive cohort study, we have to live with some model ambiguity. In fact, even if we have survey and interview data from a large cohort, the notion of reward is highly subjective. This makes comparisons between individuals at the same time problematic, much less comparisons of the same individuals at different times. This is why we chose to build the simplest model encapsulating all the important processes outlined in Moffitt’s theory and show Moffitt’s taxonomy emerges through the balance between cost and reward of imitation.

Finally, our intervention analysis showed that the two groups are not completely pre-determined, and agents can evolve into one group or the other as a result of their initial conditions and interactions with their ‘neighbors’. There are important implications that agents are not fated to become life-course-persistent or adolescence-limited. First and foremost amongst these implications is that interventions may help reduce the level of adult delinquency. In the literature, we find that most psychology and social work experts agree that interventions targeted at adolescents will not be effective, and advocate early childhood prevention at the school and community level instead [56, 57]. The Office of Juvenile Justice and Delinquency Prevention (OJJDP) Study Group on Very Young Offenders recommends integrated school and community prevention programs combining behavior management, social competence building, channeling of energy into positive recreational activities, and mentoring, [58]. In general, interventions are multimodal [59–62], and it is frequently difficult to gauge the effectiveness of intervention [63, 64]. We feel that agent-based modeling and simulation presents great opportunities for the computational testing of the efficacy of interventions in complex social problems such as juvenile delinquency. In this context it was reassuring that when we ran intervention simulations in our model, it was possible to reduce the antisocial levels of a significant fraction of agents simply by changing their network neighborhoods. The effect of this intervention is the most pronounced for borderline life-course-persistent agents. This result suggests that we should not give up on potential life-course-persistent youths, because they may be helped by various intervention strategies, although some of these may be difficult to implement in our agent-based model.
